# The Safety and Efficacy of Adjustable Postoperative Position after Pars Plana Vitrectomy for Rhegmatogenous Retinal Detachment

**DOI:** 10.1155/2017/5760173

**Published:** 2017-03-20

**Authors:** Zhong Lin, Jin Tao Sun, Rong Han Wu, Nived Moonasar, Ye Hui Zhou

**Affiliations:** ^1^The Eye Hospital, School of Ophthalmology and Optometry, Wenzhou Medical University, Wenzhou, Zhejiang, China; ^2^Department of Ophthalmology, Qingdao Economic and Technological Development Area First People's Hospital, Qingdao, Shandong, China; ^3^Ophthalmology Department, University of the West Indies, St. Augustine, Trinidad and Tobago

## Abstract

*Purpose.* To report the safety and efficacy of adjustable postoperative position for rhegmatogenous retinal detachment (RRD). *Methods.* Retrospective review of 536 consecutive RRD eyes that underwent vitrectomy surgery for retina repair from year 2008 to 2014. The retinal breaks were divided into superior, lateral (nasal, temporal, and macular), and inferior locations, according to the clock of breaks. Patients with superior and lateral break location were allowed to have facedown position or lateral decubitus position postoperatively, while patients with inferior break location were allowed to have facedown position. *Results.* 403 eyes of 400 patients were included. The mean follow-up interval was 22.7 ± 21.3 months. The overall primary retinal reattachment rate was 93.3%. There were 24 (6.0%), 273 (67.7%), and 106 (26.3%) patients with superior, lateral, and inferior break location, respectively. The primary reattachment rate was 95.8%, 92.3%, and 95.3% accordingly. After further divided the break location into subgroups as a function of duration of symptom, postoperative lens situation, number of retinal breaks, and different vitreous tamponade, the primary reattachment rates were all higher than 82%. *Conclusion.* Adjustable postoperative positioning is effective and safe for RRD repair with different break locations. Choosing postoperative position appropriately according to retinal break locations could be recommended.

## 1. Introduction

Pars plana vitrectomy (PPV) with different vitreous tamponade, including both gas and silicone oil, followed by facedown positioning for various durations, is still considered as the most standard and effective treatment procedure for rhegmatogenous retinal detachment (RRD) repair in many regions/countries [1–6]. However, the postoperative facedown positioning is an ordeal for almost all patients. Elder patients; young children; or patients with cervical spondylosis, coronary heart disease, pulmonary or bronchial disease, obesity, and other comorbidities have serious difficulties persisting in the facedown positioning. Furthermore, some rare postoperative complications, like ulnar nerve palsies, pulmonary embolism and thrombophlebitis, or decubitus, would develop after a long period of facedown position [7–9].

A decade ago, Sharma et al. reported a high primary retinal reattachment rate (81.3%) for RRD patients with inferior breaks after PPV with gas tamponade with face up or lateral check down postoperative position for 50 minutes in an hour for 7 days [3]. Martinez-Castillo et al. consecutively reported a high primary retinal reattachment rate for pseudophakic RRD patients with inferior breaks after PPV with air/gas tamponade with only 24 hours (93.3%) or even without (90–94%) postoperative facedown position [10–12]. Recently, Chen et al. reported that for RRD repair, the primary retinal reattachment rate of PPV with gas tamponade with an adjustable postoperative position (alternative upright or lateral recumbent) was as high as traditional strict facedown position (92.3% versus 89.7%) [13]. However, the safety and efficacy of adjustable postoperative position on the outcome of PPV with different ocular diseases, vitreous tamponade, and postoperative lens situation for RRD repair remain unclear. Hence, this study aims to provide further data on the safety and efficacy of adjustable position for RRD repair.

## 2. Methods

Rhegmatogenous retinal detachment (RRD) patients that underwent pars plana vitrectomy from January 2008 to December 2014 at The Eye Hospital of Wenzhou Medical University were consecutively collected. The exclusion criteria were (1) ocular penetrating trauma history or traumatic RRD, (2) previous retinal detachment repair surgery in the same eye, (3) shorter than 3 months follow-up, and (4) incomplete information on retinal break and retina reattachment postoperation or during follow-up.

A complete ocular examination was performed in each patient, including slit lamp examination, visual acuity converted to logarithm of the angle of minimal resolution (LogMAR), intraocular pressure (IOP) measurement, and fundus and peripheral retinal examination. The number, location, type, and size of retinal detachment and retinal breaks were recorded both before and during surgery. The information of breaks during the surgery was used for further analysis. The visual acuity of finger counter, hand move, light perception, and no light perception was converted to LogMAR 2, 3, 4, and 5, respectively.

All patients underwent similar surgical procedure by the same surgeon (RHW). PPV was performed using either a 23-gauge or a 20-gauge system (Accurus; Alcon Laboratories Inc., Fort Worth, TX) after retrobulbar anesthesia with a 50% mixture of 2% lidocaine and 0.75% bupivacaine. To begin the surgery, three cannulas, that is, the inferior-temporal infusion cannula and the superior-nasal and superior-temporal operation cannulas, were established. Phacoemulsification would be performed for patients with cataract (generally, phacoemulsification would be performed for patients with cataract who are being considered to impede vitrectomy operation). Then, viscoelastic substance was injected into the anterior chamber to maintain its pressure. A core vitrectomy was followed by peripheral vitrectomy using scleral indentation to remove any residual traction around the retinal breaks and anterior vitreous gel at the vitreous base. Perfluorodecalin (Huajieshi Medical Facility Limited Company, Shanghai, YZB 2671-2012) was injected into the vitreous cavity to flatten the retina. A complete fluid-air exchange was performed. Care was taken to ensure that the retina was completely reattached and that all the perfluorodecalin had been removed. Retinal breaks were surrounded by at least two rows of confluent endophotocoagulation or treated endocryotherapy (cryotherapy spot was approximately 1 mm wider than the break area). At the end of the surgery, perfluorocarbon gas (C_3_F_8_ or C_2_F_6_, Huajieshi Medical Facility Limited Company, Shanghai, YZB 3394-2011) or silicone oil (Bausch & Lomb, 5000 centistokes) was injected into the vitreous cavity, followed by intraocular lens (IOL) implantation, if needed. The main indications for silicone oil injection were large or multiple retinal breaks, or RRD combined with proliferative vitreoretinopathy, proliferative diabetes retinopathy, choroidal retinal detachment, or pathological myopia.

**Figure 1:**
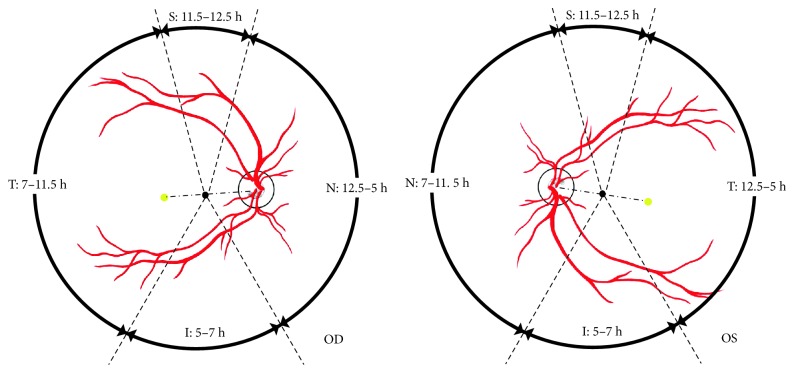
The schematic diagram showing the locations of the retinal breaks. S: superior, T: temporal, I: inferior, N: nasal, h: clock hour.

Patients were allowed to have facedown position or have alternatively facedown or lateral position, according to the location of retinal breaks, when sitting, walking, lying down, or sleeping after the surgery. The following rules were taking the right eye for example, and so on for the left eye. The locations of breaks were divided into superior (11.5 to 12.5 o'clock); lateral, that is, nasal (12.5 to 5 o'clock) and temporal (7 to 11.5 o'clock); and inferior quadrants (5 to 7 o'clock). The macular hole was also considered as a temporal break (Figure 1). The details of rules for postoperative position were as follows. Location 1 (superior break): patients with superior break(s) of either eye could alternatively choose facedown position or lateral position of either side. Location 2 (lateral break): patients with lateral break(s), for example, patients with (1) temporal break(s); (2) temporal and superior break(s); and (3) temporal and nasal with or without superior break(s) (in which the lowest break was in the temporal break; for example, 8 o'clock for the temporal break and 2 and 3 o'clock for the nasal breaks or 8 and 10 o'clock for the temporal breaks and 3 o'clock for the nasal break), could alternatively choose facedown position or left lateral position. However, if the nasal break was as low as the lowest temporal break, the patients could only choose facedown position. Location 3 (inferior break): patients with inferior break(s), with or without lateral break(s), and superior break(s) could have facedown position.

This postoperative position was not required during meals, toilet, or shower. There was no requirement for time distribution of these two alternative positions since it was chosen by the patients' own will. The daily duration and total duration of this adjustable postoperative position mainly depend on the vitreous tamponade. Generally, total duration was required to be approximately 1-2 weeks and 3 months for vitreous tamponade with gas and silicone oil, respectively. The daily duration was required to be approximately 12 hours for gas tamponade, 12 hours for the first week, and 8 hours for the later 3 weeks (if the retina was attached after follow-up examination) for silicone oil tamponade. Patients were explained and required to implement the postoperative position immediately after the surgery. During the admittance, patients were frequently visited and confirmed the postoperative position by the surgeon and other ophthalmologists. However, the patients' compliance of these postoperative positions could not be monitored strictly after discharge.

The vitreous tamponade and its relationship to the retinal breaks were carefully examined using slit lamp and indirect ophthalmoscopy several hours and 1 day after the surgery. The patients were routinely followed up at 1 week, 2 weeks, 1 month, and 3 months and then followed up as necessary. Silicone oil would be routinely removed after vitreous tamponade for 3–6 months. Additional procedures, such as photocoagulation, membrane peeling, and gas/silicone oil tamponade, would be performed when necessary during the silicone oil removal surgery. Retinal reattachment was accessed after the silicone oil was removed. The old RRD was defined as the RRD with subretinal membrane and/or thick yellowish subretinal fluid. All the information was obtained from the electronic records of the hospital.

Primary retinal reattachment rate among different retinal break locations, vitreous tamponade, and so on were compared using Fisher's exact test. Since the small sample of patients with superior retinal break, they were combined with patients with lateral break for further subgroup comparison of retinal reattachment rate. A *p* value of <0.05 was considered statistically significant. All statistical analysis was performed with Statistical Analysis System for Windows version 9.1.3 (SAS Inc., Cary, NC).

## 3. Results

536 eligible rhegmatogenous retinal detachment eyes were reviewed. 48 eyes with ocular-penetrating trauma history or traumatic RRD, 44 eyes with previous RD surgery, and 41 eyes with follow-up shorter than 3 months were excluded. Hence, 403 eyes of 400 patients (222 males, 55.5%) were finally included for further analysis. All patients were Chinese individuals aged 55.0 ± 13.9 (range 19 to 86) years old. The mean follow-up time was 22.7 ± 21.3 months. The mean duration of fresh and old RRD was 18.6 ± 17.6 days and 12.8 ± 9.2 months, respectively. More than half of the eyes (251, 62.3%) had multiple retinal breaks. Single temporal break accounted for most (135, 33.5%) of the breaks, followed by the single nasal break (42, 10.4%). Half of the macula (204, 50.6%) were involved because of the detached retina or macular hole. The retinal break(s) were categorized into 3 locations according to their location (see details in Section 2). There were 24 (6.0%), 273 (67.7%), and 106 (26.3%) eyes in location 1 to location 3, respectively (Table 1).

Single PPV were performed for the majority of the eyes (203, 50.4%), followed by PPV plus phacoemulsification plus IOL implantation (188, 46.6%), and PPV plus phacoemulsification (12, 3.0%). After the surgery, most of the eyes were pseudophakic (55.3%) or phakic (40.7%), while only 4.0% of the eyes were aphakic. The silicone oil was the major vitreous tamponade for RRD repair after the surgery (82.1%) (Table 2).

Table 3 presents the primary retinal reattachment rate in different groups. The overall primary retinal reattachment rate achieved to 93.3% (376/403). The primary reattachment rate was higher than 90% in most of the groups, except for old retinal detachment (89.3%), gas tamponade (87.5%), and macular hole group (86.1%). The primary reattachment rate was 95.8%, 92.3%, and 95.3% for superior, lateral, and inferior retinal break location, respectively. After further dividing the retinal break location into subgroups as a function of patients' gender, retinal duration, postoperative lens situation, vitreous tamponade, number of retinal breaks, and macular involvement, the primary reattachment rates were all higher than 82% (Table 4).

There were 27 out of 403 eyes (6.7%) that had reoccurred retinal detachment after primary retinal detachment repair surgery. The mean time of reoccurred retinal detachment was 108 (lower and upper quartile, 33 and 171) days after primary surgery. A second retinal detachment repair surgery was performed for all 31 eyes. The mean time between secondary surgery and last follow-up was 19.8 (lower and upper quartile, 7.5 and 48.6) months. Seven of the 27 eyes (25.9%) had a retinal reattachment after the second repair, which made the final reattachment rate reach up to 95.0%.

The IOP was increased from 10.8 ± 4.1 preoperatively to 14.3 ± 6.3 mmHg postoperatively (*p* < 0.001). There were 190 eyes (47.1%) that had IOP higher than 25 mmHg postoperatively. Among these eyes, 81.6% (155/190) and 18.4% (35/190) were tamponaded with silicone oil and gas, respectively. Besides, 173 (91.1%) and 6 (3.2%) eyes had anti-IOP eye drops and antiglaucoma surgery, respectively. Until the last follow-up, 14 eyes (14/403, 3.5%) had IOP higher than 25 mmHg. The uncorrected visual acuity (UCVA) improved from LogMAR 1.72 ± 0.97 preoperatively to 1.32 ± 0.76 one week postoperatively and 0.98 ± 0.71 at last follow-up. The best-corrected visual acuity was 1.08 ± 0.90 preoperatively and 0.71 ± 0.70 at last follow-up. However, the BCVA was only obtained in 96 eyes preoperatively and 183 eyes at last follow-up.

## 4. Discussion

More and more surgeons are trying to reduce or eliminate facedown positioning after macular hole and RRD repair surgery, in order to increase patients' comfort and compliance and decrease the potential systemic complications [3, 10–13]. Recently, Chen et al. reported that for RRD repair, the primary retinal reattachment rate of PPV with gas tamponade with an adjustable postoperative position (alternative upright or lateral recumbent) was as high as traditional strict facedown position (92.3% versus 89.7%) [13]. Furthermore, the final retinal reattachment rate were both 100% in adjustable position and in facedown position [13]. These results were inspiring for retinal surgeons and patients. However, that study excluded patients with documented previous ocular disease (other than acute RRD, previous cataract surgery, and/or refractive error), patients younger than 18 years or older than 80 years, giant retinal tear, proliferative vitreoretinopathy (PVR) of Grade C or greater, retinal detachment accompanied by choroidal detachment and/or RRD caused by macular hole in the eyes with high degree myopia (−6.00 diopter or above), duration of symptoms longer than 4 weeks, incomplete intraoperative drainage of subretinal fluid, and hypotony on the 1st day visit. Hence, the safety and efficacy of this adjustable position may be limited to certain RD patients.

The advantage of this study was that the exclusion criteria were only limited to patients with ocular penetrating trauma history or traumatic RRD, with previous retinal detachment repair surgery. To our knowledge, this study included the largest sample and most risk factors on the postoperative position after RRD repair. Hence, this study not only confirmed previous studies that postoperative facedown position was not the only choice but also indicated to popularize to more kinds of retinal detachment patients, such as retinal detachment patients with long duration, different postoperative lens statuses, silicone oil tamponade, and pathologic myopia. However, it should be mentioned that different with Chen et al., though strict facedown positions was not the only choice, the upright postoperative position which seemed to be more comfortable was not recommended. This warranted further studies.

There were several important findings in this study. First, the overall primary and secondary retinal reattachment rates were high (93% and 95%, respectively) in patients with adjustable postoperative position. The primary rate was comparable with previous reports with traditional postoperative facedown position (75–89.7%) [13–15] or adjustable position (81–94%) [3, 10–13]. In this study, though we found that the primary retinal reattachment rate was lower for the eyes with gas tamponade compared to the eyes with silicone oil tamponade (87.5% versus 94.5%), the rate was still comparable with previous studies on the eyes with gas tamponade (81–94%) [3, 10–13]. Second, more retinal breaks were categorized as superior or lateral breaks and would have more comfortable postoperative positions subsequently. Based on clinic observation, when the eyes rotated appropriately, the silicone oil or gas could give certain pressure to the retina. Hence, we shrank the inferior retinal break from the previous 4 to 8 o'clock [3, 10–12] to the current 5 to 7 o'clock. Importantly, we found that the primary retinal reattachment rate of each location was higher than 90%. Third, after further dividing the retinal break location into subgroups, the primary retinal reattachment rate was still satisfactory (higher than 80%), even in patients with inferior retinal breaks. This further improved the safety and efficacy of the adjustable postoperative position.

The IOP increased from approximately 11 mmHg preoperatively to 14 mmHg postoperatively. It was known that the IOP was generally lower in retinal detachment eyes and usually elevates in the early postoperative period after retinal detachment repair [16, 17]. However, whether this repair increases the risk of glaucoma remains controversial [17–19]. Overall, nearly half of the eyes had IOP higher than 25 mmHg postoperatively. Similar with previous reports [17], the eyes with silicone oil tamponade had a high risk to increase IOP than gas tamponade in this study (81.6% versus 18.4%). At the last follow-up, less than 5% of the eyes had IOP controlled higher than 25 mmHg. It should also be mentioned that, though only UCVA was obtained in this study, it generally increased postoperatively.

Important limitations remained in this study. First, recall bias was inevitable since this was a retrospective study. Hence, important information, such as best-corrected visual acuity and the actual postoperative position, could not be obtained completely. Second, there was no controlled group (strict facedown position). However, since the primary retinal reattachment rate was higher than 90%, and comparable with previous studies, this limitation did not seem to produce significant bias. Third, some combinations of the retinal break (such as breaks at inferior plus macular plus nasal or inferior plus macular plus superior) did not exist in this study. However, these combinations were speculated with very low incidence; this may also not have significant bias. Last, discomfort was still inevitable even with alternative postoperative positions since upright position was not recommended. Hence, further prospective, randomized, controlled studies with reduced time for facedown/lateral position even with upright positions were warranted.

In summary, choosing postoperative position appropriately according to retinal break locations could be recommended after sufficient surgery treatment, such as complete remove of vitreous traction, and sufficient endophotocoagulation. This adjustable position could improve patient compliance while it does not reduce the surgery success rate.

## Figures and Tables

**Table 1:** Patient's preoperative characteristics.

Variable	
Age (mean ± SD, year)	55.0 ± 13.9
Gender (male/female)	222/178
Follow-up time (month, mean ± SD)	22.7 ± 21.3
Duration of symptoms	
Fresh RD (*n*, mean ± SD, day)	375, 18.6 ± 17.6
Old RD (*n*, mean ± SD, month)	28, 12.8 ± 9.2
Preoperative UCBA	1.72 ± 0.97
IOP (mmHg)	10.8 ± 4.1
Macular involved (on/off)	204/199
Preoperative lens status (aphakic/phakic/pseudophakic)	3/362/38
Number of breaks (single/multiple)	152/251
Pathologic myopia (no/yes)	329/74
Location of retinal breaks	
Location 1	
S	24 (6.0)
Location 2	
T & N	177 (135/42)
T + S & N + S	25 (19/6)
T + N & T + N + S	37 (32/5)
M & M + T & M + T + S	30 (16/11/3)
M + N & M + T + N & M + T + N + S	4 (2/1/1)
Total (*n*, %)	273 (67.7)
Location 3	
I	23
I + S & I + T & I + N & I + T + N	56 (2/33/13/8)
I + T + S & I + N + S & I + T + N + S	18 (9/3/6)
I + M & I + M + T & I + M + T + N + S	9 (3/5/1)
Total (*n*, %)	106 (26.3)

SD: standard deviation; RD: retinal detachment; UCBA: uncorrected visual acuity; IOP: intraocular pressure; S: superior retinal break; T: temporal retinal break; N: nasal retinal break; I: inferior retinal break; M: macular hole.

**Table 2:** Patient's postoperative characteristics.

Variable	Number (%)
Surgery procedures	
Vitrectomy	203 (50.4)
Vitrectomy + Phaco	12 (3.0)
Vitrectomy + Phaco + IOL	188 (46.6)
Postoperative lens status	
Aphakic^∗^	16 (4.0)
Phakic	164 (40.7)
Pseudophakic	223 (55.3)
Vitreous tamponade	
Gas (C_2_F_6_ & C_3_F_8_)	72 (17.9)
Silicone oil	331 (82.1)

Phaco: phacoemulsification; IOL: intraocular lens; ^∗^3 eyes were original aphakic, 11 eyes underwent phacoemulsification without IOL implantation, and 2 eyes underwent IOL removal.

**Table 3:** Primary retinal reattachment rate in different subgroups.

	Number (%)	*p* value^∗^
Total	376 (93.3)	—
Gender		
Male	212 (94.6)	0.24
Female	164 (91.6)	
Duration of symptoms		
Fresh RD	351 (93.6)	0.42
Old RD	25 (89.3)	
Postoperative lens status		
Aphakic	15 (93.8)	0.68
Phakic	151 (92.1)	
Pseudophakic	210 (94.2)	
Vitreous tamponade		
Gas (C_2_F_6_ & C_3_F_8_)	63 (87.5)	0.038
Silicone oil	313 (94.6)	
Number of retinal breaks (single/multiple)	
Single	143 (94.1)	0.69
Multiple	233 (92.8)	
Macular hole		
No	339 (94.2)	0.055
Yes	37 (86.1)	
Pathologic myopia		
No	306 (93.0)	0.80
Yes	70 (94.6)	
Retinal break location		
Location 1 (superior)	23 (95.8)	0.65
Location 2 (lateral)	252 (92.3)	
Location 3 (inferior)	101 (95.3)	

RD: retinal detachment.

*p* value^∗^: tested by Fisher's exact test.

**Table 4:** Primary retinal reattachment rate in different retinal break location subgroups.

	Locations 1 (superior) & 2 (lateral)		Location 3 (inferior)	
	Number (%)	*p* value^∗^	Number (%)	*p* value^∗^
Gender				
Male	149 (94.3)	0.27	63 (95.5)	0.99
Female	126 (90.7)		38 (95.0)	
Duration of symptoms				
Fresh RD	256 (92.8)	0.66	95 (96.0)	0.29
Old RD	19 (90.5)		6 (85.7)	
Postoperative lens status				
Aphakic	13 (92.9)	0.24	2 (100.0)	0.68
Phakic	93 (89.4)		58 (96.7)	
Pseudophakic	169 (94.4)		41 (93.2)	
Vitreous tamponade				
Gas (C_2_F_6_ & C_3_F_8_)	34 (82.9)	0.02	29 (93.6)	0.63
Silicone oil	241 (94.1)		72 (96.0)	
Number of retinal breaks (single/multiple)				
Single	127 (94.1)	0.51	16 (94.1)	0.99
Multiple	148 (91.4)		85 (95.5)	
Macular hole				
No	246 (93.5)	0.15	93 (95.9)	0.36
Yes	29 (85.3)		8 (88.9)	
Pathologic myopia				
No	223 (92.5)	0.99	83 (94.3)	0.59
Yes	52 (92.9)		18 (100)	

RD: retinal detachment.

*p* value^∗^: tested by Fisher's exact test.
